# Sequential Total Variation Denoising for the Extraction of Fetal ECG from Single-Channel Maternal Abdominal ECG

**DOI:** 10.3390/s16071020

**Published:** 2016-07-01

**Authors:** Kwang Jin Lee, Boreom Lee

**Affiliations:** Department of Biomedical Science and Engineering (BMSE), Institute of Integrated Technology (IIT), Gwangju Institute of Science and Technology (GIST), Gwangju 61005, Korea; lightjin619@gist.ac.kr

**Keywords:** fetal ECG, abdominal ECG, total variation denoising

## Abstract

Fetal heart rate (FHR) is an important determinant of fetal health. Cardiotocography (CTG) is widely used for measuring the FHR in the clinical field. However, fetal movement and blood flow through the maternal blood vessels can critically influence Doppler ultrasound signals. Moreover, CTG is not suitable for long-term monitoring. Therefore, researchers have been developing algorithms to estimate the FHR using electrocardiograms (ECGs) from the abdomen of pregnant women. However, separating the weak fetal ECG signal from the abdominal ECG signal is a challenging problem. In this paper, we propose a method for estimating the FHR using sequential total variation denoising and compare its performance with that of other single-channel fetal ECG extraction methods via simulation using the Fetal ECG Synthetic Database (FECGSYNDB). Moreover, we used real data from PhysioNet fetal ECG databases for the evaluation of the algorithm performance. The R-peak detection rate is calculated to evaluate the performance of our algorithm. Our approach could not only separate the fetal ECG signals from the abdominal ECG signals but also accurately estimate the FHR.

## 1. Introduction

Identification of fetal distress is important in fetal monitoring during pregnancy. A noninvasive fetal monitoring method for measuring the fetal heart rate (FHR) is essential to assess the health of a fetus [1]. Cardiotocography (CTG) and fetal electrocardiogram (ECG) are common methods for monitoring the FHR [2]. The Doppler ultrasonic technique measures the FHR from the fetal heart activity using the Doppler effect. However, fetal movements and the blood flow through the maternal blood vessels always influence the Doppler ultrasound signal [3]. As the ultrasound signal includes these influences, the R-peak detection of the FHR becomes very challenging. Moreover, the mother should maintain a reclining position for one hour while the FHR data is being collected. Therefore, this approach is not suitable for long term continuous monitoring [4].

Various techniques have been proposed to extract the fetal ECG from the abdominal ECG signals of pregnant women [5,6,7]. Long-term monitoring is possible using these techniques; currently, independent component analysis (ICA) and principle component analysis (PCA) has been applied to the maternal ECG signal from the abdomen to extract the fetal ECG [7]. However, multichannel data are required for the ICA. If the number of ECG signals from the maternal abdomen is insufficient, the pure fetal ECG signals cannot be extracted using ICA [8].

On the other hand, various FHR estimation methods have been developed for single-channel data in several studies [9,10,11,12]. The template subtraction (TS) method, in particular, was commonly used for removing the maternal ECG components and extracting the fetal ECG [9,10]. However, the residual signal after removing the maternal ECG is still affected by several artifacts. This makes it difficult to find the exact location of the fetal ECG R-peaks using the TS method. The extended Kalman filter (EKF) framework has shown good performance for the extraction of the FHR [11,12,13,14]; it focuses on the cancellation of the maternal ECG. However, this method cannot extract the R-peak of the fetal ECG if the residual signal after eliminating the maternal ECG has a low-amplitude fetal ECG. Furthermore, when the QRS complexes of the maternal ECG and fetal ECG overlap, the residual signal can lose the QRS-complex segment of the fetal ECG. Therefore, the EKF framework may miss the detection of the fetal R-peak. Another estimation method is the wavelet-based approach [15]. Wavelet analysis is a time-frequency decomposition approach that can simultaneously eliminate the baseline and the high-frequency noises from the abdominal ECG and then, efficiently detect the fetal R-peaks from the filtered abdominal ECG. Although the wavelet-based approach also shows satisfactory results, the R-peak of the fetal ECG cannot be detected from the noisy residual signals after cancelling the maternal ECG.

Recently, sparse representations for signal processing have been studied for noise reduction [16,17]. Total variation denoising (TVD) has been widely used to reduce noise in image processing [18]. This approach can preserve the sharp edges of the underlying signal. Recently, Ning et al. proposed sparse derivative denoising for ECG enhancement using TVD [19]. This method can be used as a preprocessing stage for ECG analysis.

In this paper, we propose a method for measuring the FHR from the single-channel abdominal ECG signal of a pregnant woman using sequential TVD (STVD). To extract the fetal ECG, we established an abdominal ECG model and then, TVD [18] was applied twice, sequentially. Recent advanced research provided a standard methodology for benchmarking of fetal ECG extraction methods [20,21]. We followed the testing methodology to evaluate our proposed method. The remainder of this paper is organized as follows. In Section 2, the proposed STVD algorithm is described. In Section 3, we compare the performance of this method with those of three other single-channel fetal-ECG-extraction methods including the EKF and TS_PCA_ using simulation signals and real signals from medical databases [22]. To prove the importance of the second TVD step, a method in which TVD is applied only once to extract the fetal ECG, i.e., a combining TVD and TS_PCA_ (TVD + TS_PCA_) is also compared with the proposed method. Finally, a discussion about the results of our proposed method and the conclusions are presented in Section 4.

## 2. Methods

### 2.1. Preprocessing

The abdominal ECG signal is usually contaminated by baseline wandering, electromyogram (EMG) noise, and power-line noise. We applied a third-order Butterworth band-pass filter with 3-Hz and 90-Hz cutoff frequencies to remove both the baseline wandering and EMG noise [10]. A 50 or 60-Hz notch filter was used to eliminate the power-line noise [10].

### 2.2. Proposed Method: Sequential TVD with TS_PCA_

**Figure 1:**
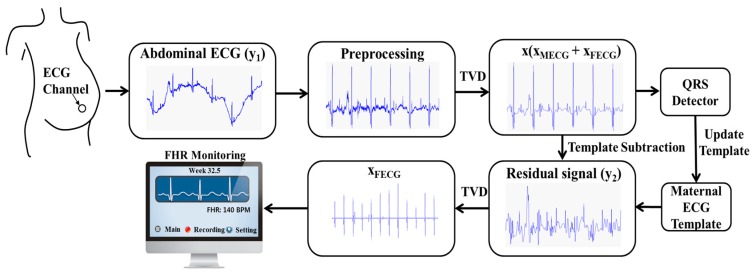
Block diagram of STVD to extract the fetal ECG from single-channel abdominal ECG.

The proposed algorithm consists of three steps. First, the abdominal ECG is filtered using TVD. Then, the maternal ECG is extracted from the abdominal ECG using the TS_PCA_ method. Finally, TVD is applied again as a cascaded process to the residual signal for estimating the fetal ECG. Figure 1 briefly illustrates the processes in our proposed algorithm.

#### 2.2.1. First TVD: Enhancing the Abdominal ECG

The maternal abdominal ECG signal $y_{1}$ is given by:

(1) $$y_{1}=x+e_{1}$$

where the vector x is a clean abdominal signal of length *N* and $e_{1}$ is the residual noise. $y_{1}$ is the contaminated measurement of x. The clean abdominal signal x is given by:

(2) $$x=x_{MECG}+x_{FECG}$$

where the vectors **x**_*MECG*_ and **x**_*FECG*_ with *N* time points represent the maternal ECG and fetal ECG signals, respectively. **x** can be extracted using the TVD problem as follows:

(3) $$x^{*}=\underset{x}{\mathrm{argmin}}\frac{1}{2}{\left\|y_{1}-x\right\|}_{2}+\lambda_{1}{\left\|D_{2}x\right\|}_{1}$$

where ${\left\|\cdot \right\|}_{n}$ is the *ℓ^n^* (*n* = 1, 2) norm in *ℝ^N^* (*N*: data length), the regularization parameter *λ_1_ > 0*, and $D_{2}$ is the second-order difference matrix. As the ECG can be approximated by piecewise linear function [18], $D_{2}$ makes the signal sparser. By increasing *λ_1_*, more weight is given to the second term that measures the fluctuation of the signal **x**. The second-order difference matrix is given by:

(4) $$D_{2}=[\begin{array}{cccccc} -1 & 2 & -1 & 0 & \cdots & 0\\ 0 & -1 & 2 & -1 & \begin{array}{c} \ddots \end{array} & \vdots \\ \vdots & \ddots & \ddots & \ddots & \ddots & 0\\ 0 & \cdots & 0 & -1 & 2 & -1 \end{array}]$$

In general, $D_{2}$ is a Toeplitz matrix of size (N−2)×N.

To extract a clean abdominal ECG, Equation (3) must be solved. However, this optimization problem cannot be solved explicitly. The Majorization–Minimization (MM) algorithm is a method for finding the solution to the optimization problem [23]. A convex function Q(x) was minimized using iteration as follows:

(5) $$Q(x)=\frac{1}{2}{\left\|y_{1}-x\right\|}_{2}^{2}+\lambda_{1}{\left\|D_{2}x\right\|}_{1}$$

(6) $$x_{\left(i+1\right)}=\arg\text{​}\underset{x}{\min}L_{i}(x),\;i=0,\;1,\;2,\;\ldots$$

$L_{i}(x)$ should be a majorizer of Q(x), that is,

(7) $$L_{i}(x)\geq Q(x),\;\forall x,$$

and $L_{i}(x)$ should satisfy Q(x) at $x_{i}$,

(8) $$L_{i}(x_{i})=Q(x_{i})$$

where $L_{i}(x)$ and $Q(x_{i})$ are a convex function. $x_{i}$ converged to the minimizer of $Q(x_{i})$ by the MM algorithm. When $L_{i}(x_{i})$ coincide with $Q(x_{i})$ at $x_{i}$, $x_{i+1}$ can be obtained by the minimizing $L_{i}(x)$ using the MM approach. Finally, we can solve the minimization problem. The MM algorithm is briefly summarized as follows:

1. Set *i* = 0, initialize $x_{0}$.
2. Choose *L_i_(***x***)* such that
  (1) $L_{i}(x)\geq Q(x),$ for all x
  (2) $L_{i}(x_{i})=Q(x_{i})$
3. Set $x_{i+1}$ as the minimizer of $L_{i}(x)$,
4. Set *i* = *i* + 1 and go back to step 2.

#### 2.2.2. TS_PCA_: Elimination of Maternal ECG

Several TS methods were implemented for the extraction of fetal ECG. Locating the maternal QRS complexes is necessary to remove the maternal ECG using TS. In this study, TS_PCA_ method was used since this showed better performance than other TS methods in the previous study [10,19]. TS_PCA_ is described in the TS_PCA_ section (see Section 2.3.3).

#### 2.2.3. Second TVD: Extracting Fetal ECG

After removing the maternal ECG segments from the abdominal ECG, we can obtain the fetal ECG signal from the residual signal. However, the fetal ECG signal will still contain the maternal ECG and noise components. Hence, additional TVD is applied to the residual signal to enhance the fetal ECG signal as shown below:

(9) $$y_{2}=x_{FECG}+e_{2}$$

(10) $$x_{FECG}^{*}=\underset{x_{FECG}}{\mathrm{argmin}}\frac{1}{2}{\left\|y_{2}-x_{FECG}\right\|}_{2}+\lambda_{2}{\left\|D_{2}x_{FECG}\right\|}_{1}$$

where the vector $e_{2}$ represents the residual artifacts including remains of the maternal ECG and noise. The same optimization process, which uses the MM approach to solve the TVD problem, is repeated. Finally, the fetal ECG R-peaks are detected from the final estimated fetal ECG.

### 2.3. Methods for Comparison

#### 2.3.1. Extended Kalman Filter

The EKF is the nonlinear version of the standard Kalman filter. When we consider a discrete-time nonlinear system with the state vector $p_{k}$ and measurement vector $q_{k}$ at time instant *k*, this model is written with nonlinear functions f(⋅) and h(⋅):

(11) $$\left\{ \begin{array}{l} p_{k+1}=f(p_{k},w_{k})\\ q_{k+1}=h(p_{k+1},v_{k+1}) \end{array}\right.$$

where the random variables $w_{k}$ and $v_{k}$ correspond to the process and measurement noises vectors, related to the process noise covariance **Q** and measurement noise covariance **R**, respectively. In this paper, we used a synthetic dynamic ECG model [24] to extract fetal ECG from the abdominal ECG [11,12,13,14]. One heartbeat ECG cycle is generally represented by the letters P, Q, R, S, and T. We used five Gaussian functions to establish one ECG signal model defined by peak amplitude ($\alpha_{i}$), width ($b_{i}$), and each center of Gaussian function ($\xi_{i}$) with respect to the PQRST sub-waveform [13]. The index *i* represents the number of Gaussian function. $p_{k}$ is defined by $\theta_{k}$ and $z_{k}$: $p_{k}={\left[\theta_{k},z_{k}\right]}^{T}$, where $\theta_{k}$ and $z_{k}$ are phase and amplitude of the maternal ECG at time instant *k*. The state equation can describe the ECG nonlinear dynamics such as the PQRST morphology by assuming a small sampling period δ and $w_{k}={\left[0,\eta_{k}\right]}^{T}$ as follows:

(12) $$f(p_{k},w_{k})⇐\left\{ \begin{array}{l} \theta_{k+1}=(\theta_{k}+\omega \delta )\mathrm{mod}(2\pi )\\ z_{k+1}=z_{k}+\eta_{k}-\sum_{i\in \{P,Q,R,S,T\}}\delta \frac{\alpha_{i}\omega }{b_{i}^{2}}(\theta_{k}-\xi_{i})\exp\left(-\frac{{\left(\theta_{k}-\xi_{i}\right)}^{2}}{2b_{i}^{2}}\right) \end{array}\right.$$

where $\eta_{k}$ is a random additive noise. Here, the phase $\theta_{k}$ is used as an additional observation [13]. The R-peak of ECG is set to be located at $\theta_{k}=0$, and the ECG segments between two consecutive R-peaks are assumed to have a phase between 0 and 2π [13]. Consequentially, the observed phase $\varphi_{k}$ can be determined by detected R-peaks through $\varphi_{k+1}=\theta_{k+1}+u_{k+1}$. $q_{k+1}=h(p_{k+1},v_{k+1})$ is finally expressed as $q_{k+1}=p_{k+1}+v_{k+1}$, where $q_{k+1}={\left[\varphi_{k+1},s_{k+1}\right]}^{T}$ and $v_{k+1}={\left[u_{k+1},v_{k+1}\right]}^{T}$ with $s_{k+1}=z_{k+1}+v_{k+1}$. $s_{k+1}$ represents the observed amplitude. The maternal ECGs can be estimated by consecutive EKF steps. The first EKF extracts the maternal ECG from the abdominal ECG. After subtracting the maternal ECG from the abdominal ECG, the second EKF estimate fetal ECG from the residual signal.

#### 2.3.2. TVD + TS_PCA_

The process of the TVD + TS_PCA_ is similar to the STVD method. This approach first filters the abdominal ECG signal using TVD and then applies TS_PCA_ to filtered abdominal ECG signal to eliminate the maternal ECG. After applying TS_PCA_, fetal R-peak detection is performed on the residual signal. In the TVD + TS_PCA_, *λ* was set to the same value as the *λ*_1_ in the STVD approach.

#### 2.3.3. TS_PCA_

TS_PCA_ was introduced by Kanjilal et al. [25]. The design matrix X was built with several rows in which each row corresponds to one maternal ECG cycle with all R-peaks lying at the same column as follows:

(13) $$X=\left[\begin{array}{ccc} x_{11} & \cdots & x_{1n}\\ \vdots & \ddots & \vdots \\ x_{m1} & \cdots & x_{mn} \end{array}\right]$$

where *m* and *n* represents the number of maternal ECG cycles and sample sizes. The principle components (PCs) of X can be obtained using singular value decomposition. The PCs related to maternal ECG can be subtracted from the overall cycles as follows:

(14) $$X_{r}=X-\sum_{j=1}^{p}w_{j}w_{j}^{T}X$$

where *p* is the number of PCs and $w_{j}$ is the direction vector of the *j*-th PC. The residual signal $X_{r}$ is then the fetal ECG. This method needs to update the design matrix.

### 2.4. Data

There is no standard tool for the evaluation of the single-channel fetal ECG recording [11]; simulations are required to provide quantitative assessments for the proposed method. The *fecgsyn*, a non-invasive fetal ECG model simulator [26], can generate several types of maternal and fetal ECG mixed signals. Recently, Andreotti et al. developed the Fetal ECG Synthetic Database (FECGSYNDB) using the *fecgsyn* for benchmarking of fetal ECG extraction and detection algorithms [20]. This database includes 1750 realistic simulated abdominal ECG signal with 34 channels (2 channels: maternal ECG, 32 channels: abdominal ECG), generated at a 250 Hz sampling frequency for 5 min. The simulation signals were divided into seven cases with five signal-to-noise ratio (SNR) levels (0, 3, 6, 9 and 12 dB) and 10 different maternal and fetal heart dipole’s arrangements. Each simulation signal was generated five times for statistical purposes. Table 1 represents several cases of simulation signal. A recent work selected 8 channels (1, 8, 11, 14, 19, 22, 25 and 32) among 34 channels for evaluating algorithms [20]. We also used the same channels.

**Figure 2:**
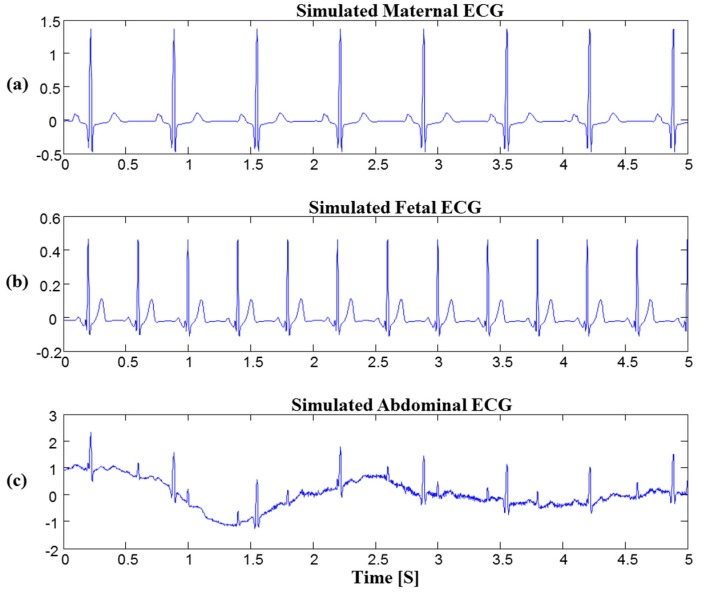
Simulated abdominal ECG using the *fecgsyn*. (**a**) Simulated maternal ECG; (**b**) Simulated fetal ECG; (**c**) Simulated abdominal ECG mixed with fetal ECG and artifacts.

Figure 2a shows the simulated maternal ECG. The fetal ECG was also constructed using the simulator (Figure 2b). The simulated fetal ECG was added to the simulated maternal ECG along with motion and EMG artifacts produced by the simulator to generate the final simulated signal (Figure 2c). To verify the efficiency of the proposed method on real data, we used PhysioNet fetal ECG databases with varying SNRs [10,21]. The database is the 2013 PhysioNet/Computing in Cardiology Challenge Database (PCDB) [22]. PCDB consisted of three data sets (set-a, set-b and set-c). We used set-a for the assessment of extraction algorithms since reference fetal QRS locations were offered. This database contained 1-min abdominal ECGs from four abdominal ECG channels at 1000 Hz. This database includes 75 datasets. However, records a33, a38, a47, a52, a54, a71, and a74 have inaccurate fetal QRS annotations. Therefore, we excluded these data and used a total of 68 datasets.

For the best channel selection, we used beat comparison measure (BCM) in each database [10]. BCM scans the maternal QRS position already removed along the residual signal, and then selects the channel in which the amplitudes of maternal QRS segments are the lowest. After selecting the best channel, the extraction fetal ECG procedure was performed. When EKF and TS_PCA_ were applied for the extraction of fetal ECG, we used open-source code in the FECGSYN toolbox [26].

### 2.5. Evaluation and Detection of Fetal ECG R-Peak

To analyze the simulation results, we detected the fetal ECG R-peaks to compare the four techniques according to the sensitivity (Se), positive predictive value (PPV) and the *F*_1_-measure. The formulae for Se, PPV, and *F*_1_ are as follows [20]:

(15) $$Se=\frac{TP}{TP+FN}$$

(16) $$PPV=\frac{TP}{TP+FP}$$

(17) $$F_{1}=2\cdot \frac{PPV\cdot Se}{PPV+Se}=\frac{2\cdot TP}{2\cdot TP+FN+FP}$$

where TP, FP, and FN denote the true positive rate, false positive rate, and false negative rate, respectively. TP indicates that true fetal ECG R-peaks were detected. FP indicates that an artifact was detected as a true fetal ECG R-peak, while FN indicates false R-peak misdetection. In addition, Se indicates the ability to detect the R-peak, and PPV provides the probability that the true fetal ECG R-peaks are detected. *F*_1_ indicates the overall probability that the fetal ECG R-peaks are detected correctly. The acceptance interval between detection and reference location of fetal R-peak was defined to be ±50 ms [10,20]. For fetal R-peak detection, many detection algorithms were offered as open-sources. Since the detection performance varies depending on algorithms, we selected the adapted version of the Pan and Tompkins algorithm which is commonly used for ECG R-peak detection [10,20]. The FECGSYN toolbox also includes this source code. Moreover, the mean absolute error (MAE) of the absolute time difference between the reference fetal R-peak location and detected fetal R-peak location was calculated to compare the four methods—STVD, EKF, TVD + TS_PCA_, and TS_PCA_, in diverse situations. MAE is expressed as:

(18) $$MAE=\frac{1}{N_{TP}}\cdot \sum_{i=1}^{N_{TP}}\left|a_{i}-{\hat{a}}_{i}\right|$$

where $a_{i}$ and ${\hat{a}}_{i}$ are the reference R-peak time location and detected R-peak time location. *N_TP_* is the total number of TP.

## 3. Results

### 3.1. Parameter Optimization

**Figure 3:**
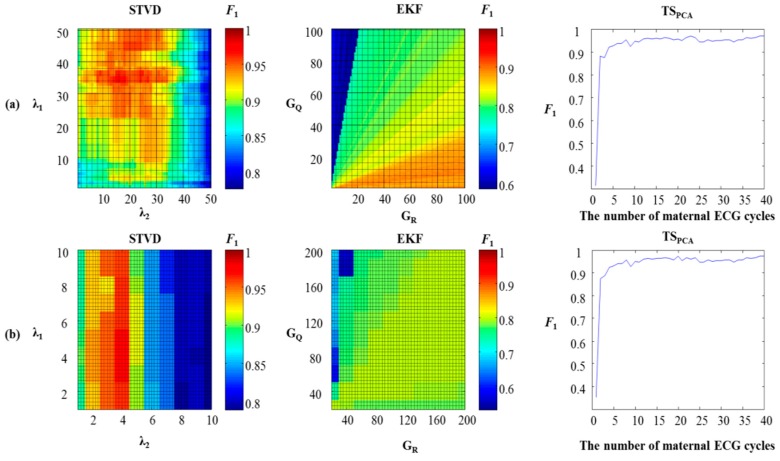
Optimal parameters search performed according to F_1_. (**a**) FECGSYNDB (0 dB); (**b**) set-a database.

Grid search was performed to find optimal parameters for STVD and EKF. On the other hand, we chose the number of maternal ECG cycles with the highest F_1_ to build the maternal ECG template for TS_PCA_. Figure 3 shows heat map and performance change with different numbers of maternal ECG cycles for each database (simulation: FECGSYNDB, real data: set-a). In this study, we multiplied covariance matrix Q and R by their gains to obtain the best performance for EFK [10]. Two gains were denoted as G_Q_ and G_R_, respectively. We found optimized G_Q_ and G_R_ values using grid search. In TS_PCA_ case, the number of PCs and the number of maternal ECG cycles influence on the performance of TS_PCA_. When the number of PCs was set to 2, we got the best result for all databases. Therefore, we searched for only the optimal number of maternal ECG cycles. Table 2 illustrates optimal parameters of each algorithm for different databases. For set-a, optimal parameters of EKF and TS_PCA_ had the same values as previous work [10].

### 3.2. Simulation

**Figure 4:**
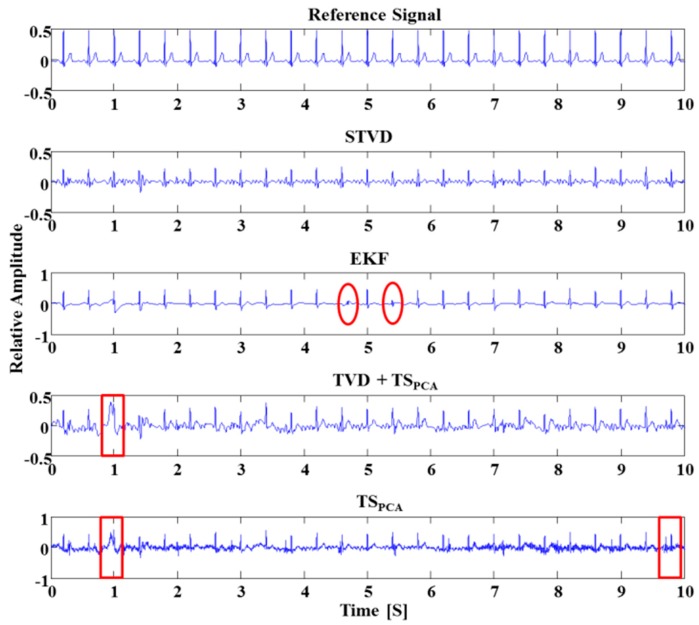
Extracting fetal ECG from the simulated abdominal ECG signal using four different methods. EKF sometimes eliminates the R-peaks of fetal ECG (red circles). Considerable noise remains after the extraction of fetal ECG by TVD + TS_PCA_ and TS_PCA_ methods (red boxes).

Realistic simulated signals were used to assess the performance of the STVD method for comparison with the performances of the other methods. The extraction of the fetal ECG signals by several algorithms is shown in Figure 4. As shown in Figure 4, the fetal ECG signals extracted by TVD + TS_PCA_ and TS_PCA_ are still influenced by artifacts. That is, as TVD + TS_PCA_ and TS_PCA_ had considerable amounts of remaining noise, it was difficult to find the exact R-peak (see red boxes in Figure 4). Moreover, the EKF could not extract the R-peaks of the fetal ECG occasionally (see red circles in Figure 4). However, the STVD method could extract all R-peaks accurately in Figure 4.

Table 3 and Table 4 show various results for evaluating the performance of each algorithm. The STVD method performs much better than other three algorithms with higher *F*_1_ for all cases. Especially, STVD has the highest *F*_1_ in uterine contraction (case 3) and ectopic beats (case 4) cases. However, the MAE results of STVD are higher than EKF method (STVD: 3.9 ms, EKF: 3.8 ms).

**Figure 5:**
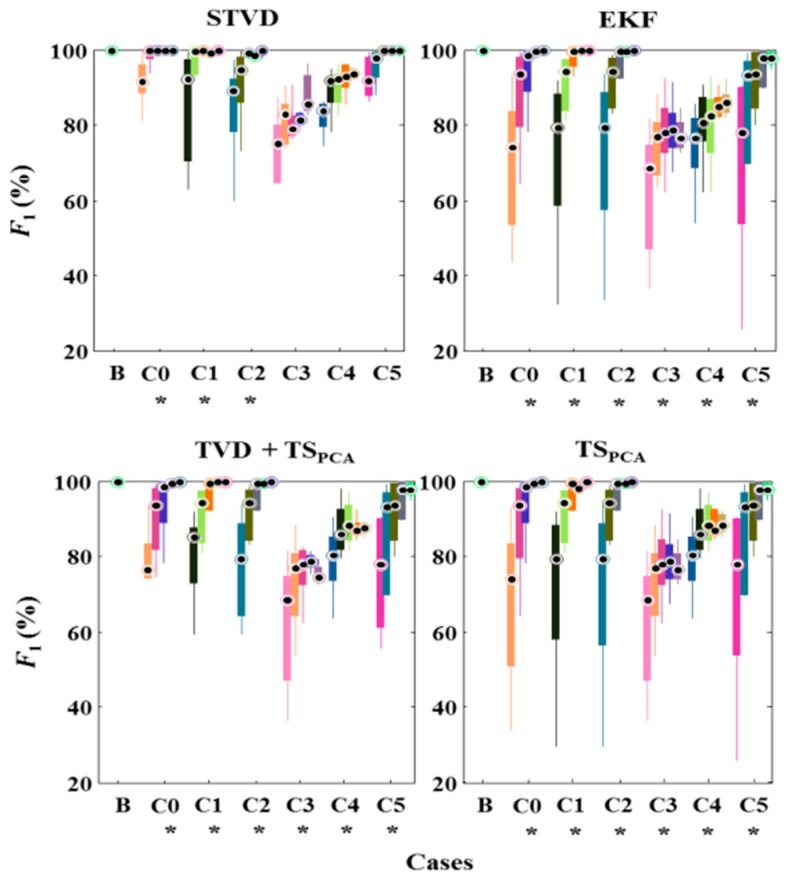
Comparison of *F*_1_ results obtained from STVD, EKF, TVD + TS_PCA_, and TS_PCA_ for each case. One case includes five box plots, one for each SNR level (0, 3, 6, 9, 12 dB); * indicates *p* < 0.05, and B is baseline. C0–C5 means 5 cases in Table 1.

**Figure 6:**
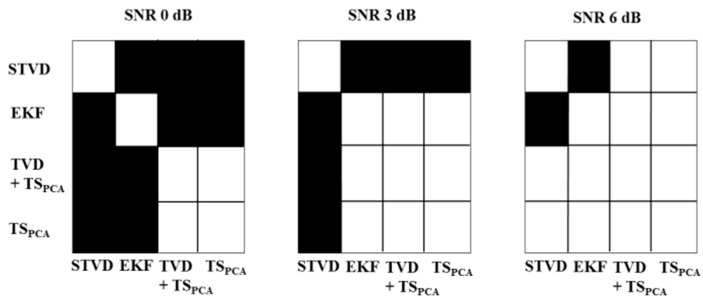
Results of the post hoc analysis using the Sign test over single-channel fetal ECG extraction methods. The post hoc analysis was performed regarding *F*_1_. Black square refers to significant differences (*p* < 0.05) and white means that there is no significant difference (*p* > 0.05). Results of SNR 9 and 12 dB are excluded because of no significant differences across methods.

Figure 5 shows results of *F*_1_ after applying noninvasive fetal ECG extraction methods in various cases with different SNR levels. We used a Kruskal-Wallis test to identify a significant effect of the SNR [19]. In particular, this test was meant to determine whether there is a statistically significant difference of *F*_1_ value according to different SNR levels. If *p*-values are smaller than 0.05, it means that the performance of extraction methods is influenced by SNR levels. As shown in Figure 5, EKF, TVD + TS_PCA_, and TS_PCA_ are vulnerable to high noise levels (*p* < 0.05), while STVD shows significant noise robustness as in case 3, 4, and 5. Furthermore, we performed a post hoc analysis using Sign test for the assessment of paired differences between fetal ECG extraction methods (see Figure 6) [20]. Since the significant differences could not be identified, we excluded plots for SNR 9 and 12 dB in Figure 6. Figure 6 illustrates that results of STVD are significantly different from other methods for low SNR 0 and 3 dB (*p* < 0.05).

### 3.3. PhysioNet Database

**Figure 7:**
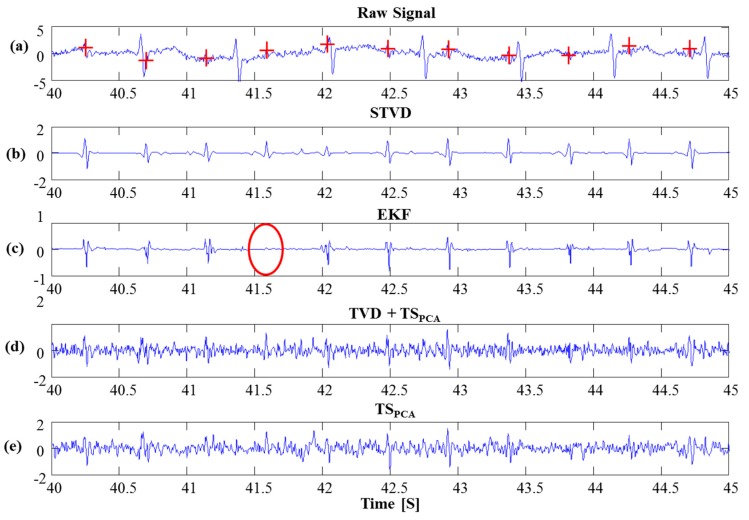
Comparison of fetal ECG extraction by STVD, EKF, TVD + TS_PCA_ and TS_PCA_ using set-a (data ‘a12’). The EKF might lose the R-peak of the fetal ECG (red circle). Red cross indicates reference fetal QRS locations (**a**) Raw Signal; (**b**) STVD; (**c**) EKF; (**d**) TVD + TS_PCA_; (**e**) TS_PCA_.

**Figure 8:**
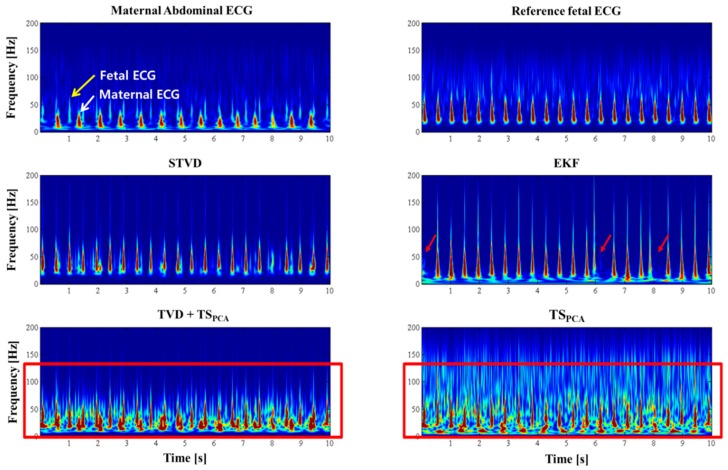
Time-frequency representation of extracted fetal ECG using the four different methods for data ‘a01’ from set-a. EKF could not detect the fetal ECG several times (red arrows). TVD + TS_PCA_ and TS_PCA_ still contain the spectral contents of artifacts (red boxes).

STVD was also applied to PhysioNet databases, and the estimated fetal ECG signals for set-a are shown in Figure 7. As shown in Figure 7c, the EKF might remove the fetal ECG from set-a dataset (see red circle). Moreover, the residual signals of the TVD + TS_PCA_ and TS_PCA_ methods are still influenced by artifacts (see Figure 7d,e). Meanwhile, we have confirmed from the set-a data that our proposed method can extract the fetal ECG accurately (see Figure 7b). Table 5 presents the results of the comparison of our proposed method with the other three methods for set-a. STVD presents higher *F*_1_ scores (*F*_1_: 89.9%) than the other methods (EKF: 81.1%, TVD + TS_PCA_: 87.4%, TS_PCA_: 86.7%). Moreover, this dataset generally has lower SNR; hence, TVD + TS_PCA_ and TS_PCA_ showed worse performances than STVD. In addition, the time–frequency presentation using the Stockwell-transform [27] in Figure 8 demonstrates that the EKF could extract the fetal ECG for set-a. However, we can confirm from Figure 8 that the EKF might detect incorrect fetal ECG R-peaks (see red arrows). Only the STVD could extract all the R-peaks of the fetal ECGs exactly in Figure 8. As can be observed in Figure 8, the spectral content of artifacts still remains in the fetal ECG signal extracted via TVD + TS_PCA_ and TS_PCA_, while STVD could isolate the spectral contents in the fetal ECG.

## 4. Discussion and Conclusions

In this paper, we proposed sequential total variation denoising (STVD) for fetal electrocardiogram (ECG) extraction from the abdominal ECGs of pregnant women. In this method, two total variation denoising (TVD) steps were used sequentially. The first TVD reduced the noise components of the abdominal ECG for effective cancellation of the maternal ECG by template subtraction (TS_PCA_) and then the second TVD enhanced the fetal ECG component. The extended Kalman filter (EKF) framework [11,12,13,14], TS_PCA_ [10], and combined TVD with TS_PCA_ (TVD + TS_PCA_) methods were compared with our proposed method. TS_PCA_ has been widely used for extracting the fetal ECG from single-channel abdominal ECGs. However, the results from simulation and real datasets showed that the TS_PCA_ method was not suitable for noisy abdominal ECGs. The performance of the TS_PCA_ approach heavily depends on accurate detection of maternal ECG R-peaks. When accurate maternal R-peak cannot be detected because of noise, the TS_PCA_ method cannot remove maternal ECG segments. Thus, TS_PCA_ showed poor performance for low SNR levels in the simulation. Moreover, the residual signal, after extracting the maternal ECG from the noisy abdominal ECG using TS_PCA_, was still noisy; this makes it difficult to confirm the fetal ECG.

**Figure 9:**
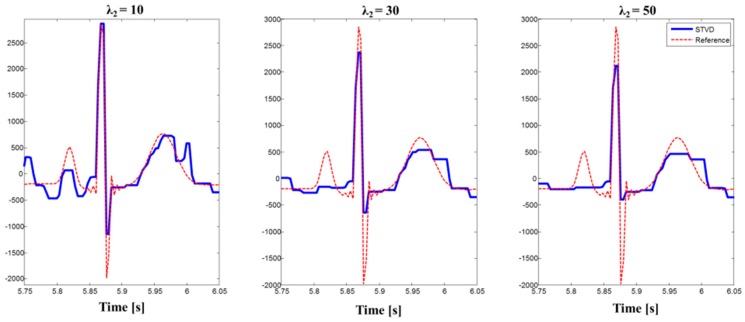
Morphological changes of the STVD extracted fetal ECG according to *λ*_2_ for simulation data ‘sub01_snr09dB_l1_c0’.

Recently, several studies have employed the EKF framework to extract the fetal ECG [11,12,13,14] as the EKF demonstrates good performance. The drawbacks of the EKF framework were shown by the simulation and real dataset. When the R-peaks of the fetal ECG and maternal ECG overlapped, it was difficult for the EKF to distinguish the fetal ECG from the maternal ECG (see Figure 4 and Figure 8) [11]. Therefore, the fetal ECG was also eliminated during the maternal ECG cancellation. Furthermore, if the amplitude of the fetal ECG was small, this method could not detect the true fetal ECG peaks (see Figure 7). As the EKF framework requires strong assumptions to establish a dynamic model, it requires reliable prior information about the fetal ECG R-peaks [28]. The EKF method showed worse performance than all other approaches for simulation and a real dataset (see Table 3 and Table 5). As the EKF approach removed the fetal ECG R-peak during the process of maternal ECG cancellation, the number of FNs increased for the two real datasets. Meanwhile, STVD could detect true fetal ECG R-peaks correctly because of the fetal ECG enhancement by the additional TVD step [18]. Therefore, STVD had higher Se values than other methods. However, EKF had the best results for MAE. This is because that EKF method estimated fetal ECG R-peaks using prior information about the fetal ECG R-peaks. On the other hand, the morphology of fetal ECG was distorted by TVD, even though STVD performed better to extract fetal ECG R-peaks from single-channel abdominal ECG. Figure 9 shows the morphological changes of fetal ECG extracted using STVD with different values of *λ*_2_. We can confirm from Figure 9 that the amplitude of PQRST waves was reduced by the TVD method. Furthermore, location of fetal R-peaks was also distorted. Therefore, MAE results of STVD had higher than EKF. However, if we can find suitable regularization parameters, STVD can extract fetal ECG with minimal fetal ECG signal distortion (See Figure 9).

Finally, to verify the importance of the second TVD step, we compared STVD with the combination of TVD and TS_PCA_. As shown in Table 3 and Table 4, TVD + TS_PCA_ showed better performance than TS_PCA_, since the TVD step could enhance the abdominal ECG. After the cancellation of the maternal ECG segments, the residual signal was less influenced by artifacts than the residual signal produced by the TS_PCA_ method. However, TVD + TS_PCA_ could not enhance the fetal ECG adequately because of its residual noise. In contrast, the STVD method could extract the fetal ECG by means of a second TVD step. The ECG waveform generally includes spike peaks. We could establish the ECG model as an approximate piecewise linear model. As the ℓ_1_ norm term of the TVD problem measures the fluctuation of the signal, TVD could preserve the signal with high total variation, such as a QRS complex. This characteristic enhanced the fetal ECG R-peaks [19,29]. Thus, STVD showed much better performance than the TVD + TS_PCA_ for both simulations and real data. Moreover, the *F*_1_ of STVD was higher than that of TVD + TS_PCA_. Overall, STVD could effectively detect true fetal ECG R-peaks and decrease the error rate of detection. Altogether, the results of this study suggest that the STVD is a promising tool for monitoring the FHR using single-channel abdominal ECGs of pregnant women. Therefore, our proposed method would help in the continuous detection of changes in the normal FHR pattern. Moreover, one of the issues for FHR monitoring is the real-time implementation. Since we already recognized low computational load in our approach, we plan to develop a real-time FHR monitoring system for future work.

## Figures and Tables

**Table 1:** Generating simulation signal for seven different physiological events.

Case	Description
Baseline (B)	Abdominal ECG without any noise or events (maternal ECG + fetal ECG)
Case 0 (C0)	Baseline (no events) + noise
Case 1 (C1)	Fetal movement noise + noise
Case 2 (C2)	Acceleration or deceleration of maternal and fetal heart rate + noise
Case 3 (C3)	Uterine contraction + noise
Case 4 (C4)	Ectopic beats of maternal and fetal + noise
Case 5 (C5)	Twin + noise

Noise includes maternal movement and EMG artifacts.

**Table 2:** Optimal parameters of each extraction method for different databases.

Method	Parameters	FECGSYNDB					Set-a
		0 dB	3 dB	6 dB	9 dB	12 dB	
STVD	λ_1_	34	12	4	1	1	4
	λ_2_	16	9	2	1	1	3
EKF	G_Q_	10	5	2	1	1	5
	G_R_	84	75	10	1	1	100
TS_PCA_	The number of maternal ECG cycles	23	23	15	14	14	20

**Table 3:** Evaluation results on *F*_1_ for FECGSYNDB, shown as median (interquartile range, %).

Method	Baseline	Case 0	Case 1	Case 2	Case 3	Case 4	Case 5	Total
STVD	100 (0.0)	98.4 (4.3)	99.4 (8.9)	98.5 (9.5)	80.2 (11.5)	90.6 (5.9)	97.5 (4.2)	97.2 (9.9)
EKF	100 (0.2)	98.1 (9.9)	98.8 (8.2)	98.2 (9.6)	77.8 (10.0)	83.4 (7.3)	94.5 (24.8)	94.5 (19.8)
TVD + TS_PCA_	100 (0.0)	97.8 (8.7)	99.4 (5.1)	98.1 (8.0)	78.7 (10.2)	88.6 (17.2)	95.9 (16.8)	96.1 (24.8)
TS_PCA_	100 (0.0)	98.3 (9.9)	99.2 (7.6)	98.7 (9.3)	77.5 (10.2)	88.4 (10.8)	94.9 (23.2)	96 (18.5)

**Table 4:** Evaluation results on MAE for FECGSYNDB shown as median (interquartile range, ms).

Method	Baseline	Case 0	Case 1	Case 2	Case 3	Case 4	Case 5	Total
STVD	3.3 (2.0)	3.6 (1.9)	3.7 (0.2)	3.2 (1.3)	4.5 (0.4)	3.2 (3.6)	3.3 (3.5)	3.9 (2.2)
EKF	4.5 (6.3)	3.8 (4.4)	3.1 (0.9)	3.1 (1.1)	3.9 (1.6)	4.8 (1.4)	3.5 (3.7)	3.8 (2.7)
TVD + TS_PCA_	3.4 (3.6)	5.2 (3.5)	5.1 (2.8)	5.5 (3.3)	4.2 (3.6)	3.4 (2.0)	3.8 (2.7)	4.2 (2.1)
TS_PCA_	4 (2.0)	4.2 (2.1)	4.2 (0.6)	4.1 (0.8)	4.7 (1.1)	5.6 (0.8)	4.3 (1.8)	4.4 (1.6)

**Table 5:** Evaluation results on fetal R-peak detection for set-a.

Method	TP	FN	FP	Se (%)	PPV (%)	*F*_1_ (%)	*MAE (ms)*
STVD	9162	946	1072	90.5	89.4	89.9	9.3
EKF	7989	1719	1984	82.2	80.1	81.1	7.9
TVD + TS_PCA_	8519	1189	1246	87.7	87.2	87.4	16.4
TS_PCA_	8475	1233	1367	87.2	86.1	86.7	14.3

Total 68 datasets were used (record a33, a38, a47, a52, a54, a71, and a74 were excluded).

## Abbreviations

The following abbreviations are used in this manuscript:

FHR	Fetal Heart Rate
ECG	Electrocardiograms
STVD	Sequential total variation denoising
TVD	Total variation denoising
ICA	Independent component analysis
PCA	Principle component analysis
TS	Template subtraction
EKF	Extended Kalman filter
MAE	Mean absolute error
Se	Sensitivity
PPV	Positive predictive value
SNR	Signal-to-noise ratio
